# From pelvic radiation to social isolation: a qualitative study of survivors’ experiences of chronic bowel symptoms after pelvic radiotherapy

**DOI:** 10.1007/s11764-023-01527-6

**Published:** 2024-01-06

**Authors:** A Biran, C Dobson, C Rees, R Brooks-Pearson, A Cunliffe, L Durrant, J Hancock, H Ludlow, L Neilson, A Wilson, L Sharp

**Affiliations:** 1 Population Health Sciences Institute, Newcastle University, Newcastle Upon Tyne, UK; 2 Northern Centre for Cancer Care, The Newcastle Upon Tyne Hospitals NHS Foundation Trust, Newcastle Upon Tyne, UK; 3 South East London Cancer Alliance, London, UK; 4 Somerset NHS Foundation Trust, Taunton, UK; 5 North Tees and Hartlepool NHS Foundation Trust, Stockton-On-Tees, UK; 6 Cardiff & Vale University Health Board, Cardiff, UK; 7 Department of Gastroenterology, South Tyneside and Sunderland NHS Foundation Trust, South Shields, UK; 8 The Royal Marsden NHS Foundation Trust, London, UK

**Keywords:** Pelvic radiotherapy, Pelvic radiation disease, Chronic bowel symptoms, Qualitative study, Late-effects

## Abstract

**Purpose:**

We explored survivors’ experiences of chronic bowel symptoms following pelvic radiotherapy, strategies employed in living with these symptoms, effects on daily activities, and roles at home and in the workplace.

**Methods:**

Semi-structured interviews were conducted with 28 individuals (10 gynaecological, 14 prostate, four anal/rectal cancer survivors) who had completed pelvic radiotherapy at least six months prior to data collection and who had experience of bowel symptoms during this post-treatment period. Reflexive thematic analysis was undertaken.

**Results:**

We propose four themes describing a process leading from experience of symptoms to withdrawal from activities and roles. These are (1) losing control (the experience of unintended anal leakage or discharge); (2) experiencing embarrassment and fear (the experience of embarrassment or fear of embarrassment as a result of discharge becoming public); (3) managing and reacting (acting to reduce the likelihood of discharge or to prevent this becoming public); and (4) restriction and withdrawal (avoiding specific activities or situations so as to reduce or remove the risk of embarrassment). Returning to the workplace presented additional challenges across these themes.

**Conclusions:**

Impacts of chronic bowel symptoms can be severe. Survivors employ a variety of methods and strategies in living with their symptoms. Some of these support continued role fulfilment but some constitute a withdrawal from pre-treatment roles. Current healthcare provision and statutory protections fail to fully meet needs following pelvic radiotherapy.

Implications for cancer survivors.

There is a need to develop and implement evidence-based services and supported self-management programmes for survivors experiencing chronic bowel problems post-radiotherapy.

**Supplementary Information:**

The online version contains supplementary material available at 10.1007/s11764-023-01527-6.

## Introduction

Pelvic radiotherapy is used to treat a range of cancers including prostate, endometrial, and cervical. Survival rates for these cancers have improved markedly [1], at least in part due to advances in treatment. This means the number of people living with and beyond cancer is growing. However, exposure to radiation can damage surrounding tissue leading to chronic symptoms which may arise and/or persist years or even decades after exposure [2]. Collectively these symptoms, which include bowel symptoms, have been termed pelvic radiation disease [3].

The bowel symptoms that people treated with pelvic radiation may experience are wide ranging and include urgency, frequency, bleeding, incontinence, mucous discharge, diarrhoea, excess wind, constipation, and pain. Questionnaire-based studies have reported varied prevalence of these symptoms and, perhaps more importantly, an associated decrease in overall quality of life (QoL) and across various QoL domains (e.g. [4–6]). However, few studies have used qualitative methods to explore survivors’ experiences of living with these bowel symptoms.

In the United Kingdom (UK), Ludlow [7] interviewed nine survivors recruited through a specialist cancer late-effects clinic and identified stigma associated with faecal incontinence as their dominant experience, highlighting the importance of support from close friends and partners. Krook [8] interviewed 10 Swedish women experiencing faecal incontinence after pelvic radiotherapy and used a phenomenological analysis to explore the meanings ascribed to their experiences. They described respondents’ lives as lived ‘in limbo’, unable to participate in activities in the way they had pre-cancer because of uncertainty about their bowel control. Peden-McAlpine [9] interviewed women living with faecal incontinence (FI), though not necessarily following cancer treatment, and found the workplace was reported by participants as the most complicated situation within which to deal with their FI symptoms.

For some people, the impact of bowel, and some of the other pelvic symptoms, may be mitigated with treatment and support. For example, a range of strategies has been set out in the recent UK *Best Practice Pathway for Pelvic Radiation Disease* [10]. However, there have been suggestions that many patients may struggle to obtain adequate attention for their symptoms from health practitioners [7] and fall through gaps in service provision. Self-management encompasses actions that people can take themselves to actively support their rehabilitation following diagnosis with a chronic condition, such as cancer (see e.g. [11]). However, little is known about the extent to which those with chronic bowel symptoms engage in self-management or how helpful they find this.

Extending the existing knowledge-base relating to chronic bowel symptoms is important to underpin advocacy, service planning, and support for survivors with these symptoms. The objectives of this qualitative study were to explore survivors’ experiences of chronic bowel symptoms, the strategies employed for self-management of these symptoms, and the combined impact of symptoms and strategies on daily activities and work.

## Methods

### Sampling and recruitment

Participants were recruited in the UK through cancer charities and National Health Service (NHS) hospitals. At our request, four cancer charities circulated study details through their mailing lists. Interested individuals contacted the research team by email and were sent an expression of interest form. Five NHS hospital Trusts collaborated on the study. Two of these offered a late-effects clinic and three provided routine hospital care after pelvic radiotherapy. Sites identified eligible patients and provided them with a recruitment pack comprising an information sheet and expression of interest form, either by post or in person. Interested individuals contacted by either route returned a completed expression of interest form to the study team. On receipt of this, these individuals were contacted by email or phone to arrange for an interview.

Individuals were eligible to take part in the study if they had been treated for a primary cancer of the prostate, bladder, cervix, endometrium rectum, or anus; had undergone pelvic radiotherapy six months or more prior to recruitment; and had experience of one or more bowel symptoms during the post-treatment period. Individuals were excluded from the study if they were awaiting bowel surgery or had received surgery in the past six months and/or were receiving treatment for a recurrence of their cancer, and/or were undergoing palliative care and or were judged by their clinical team to have psychological or communication problems or language barriers that would make it inappropriate or difficult for them to participate in an interview in English (we lacked translation capacity for non-English speaking patients). Information about the experience of bowel symptoms prior to radiotherapy was not collected or used as an exclusion criterion.

Guided by recommendations [12] we anticipated that a sample size in the region of 25–30 individuals would allow us to reach a point where further interviews would add little by way of new information. Recruitment started first through cancer charities; all eligible volunteers were interviewed. As the majority of these were women, to achieve more balanced representation, participating NHS sites were asked to prioritise recruitment of male patients.

### Data collection

Data were collected through semi-structured interviews. The main topics explored in the interviews were current symptoms and their history, self-management, support, impact on daily life, information seeking, and provision of and interaction with health services. Interviews followed a topic guide with flexibility to allow changes in the order of questions and the exploration of new topics if they arose. The topic guide was drafted by one author (AB) and was reviewed and revised by co-authors. The revised guide was reviewed by three individuals who had undergone pelvic radiotherapy and finalised. Interviews lasted up to 90 minutes and were conducted remotely, either by phone or online using Zoom or Teams. All interviews were conducted by AB, a trained and experienced qualitative researcher who had no prior relationship with participants.

Participants were offered a shopping voucher post-interview, to thank them for their time.

### Data handling and analysis

Interviews were digitally recorded, and recordings were transcribed and pseudonymised. After familiarisation with the transcripts, these were imported to NVivo 12 software and coded and a reflexive thematic analysis undertaken [13]. Initial coding of early interview transcripts used a coding framework derived from the topic guide and developed by the wider team. Three early transcripts were coded by two authors (AB and CD) to check the usefulness of the coding framework, and the framework revised accordingly. The coding framework was then applied to the remainder of the transcripts. Additional codes, themes, and subthemes were proposed by one of the authors (AB) based on repeated reading of the whole dataset. These were discussed with two co-authors (CD and LS) and agreed as plausible and useful organising constructs or rejected. As new organising constructs were developed, previously coded transcripts were reanalysed to look for instances of these. The focus of our analysis was the data relating to bowel symptoms, their impact on daily life, and the strategies used by individuals to self-manage, control, and live with these symptoms. From these data, we proposed (see the “Results” section) an organising framework comprising four themes. We then applied this framework specifically to experiences in the workplace, a topic which was probed at interview because of previous suggestions that this may be a particularly challenging situation in which to manage bowel symptoms (e.g. [9]).

Themes are reported with illustrative quotes (together with the participant’s sex, age, cancer site, symptoms experienced at recruitment, and study identification (ID)) followed by a description (also reported with illustrative quotes) of participants’ experiences across these themes in the context of work and the workplace. Additional quotes are presented in Online Resource 1.

## Findings

### Population sample characteristics

The population sample comprised 28 participants of whom 50% were female (Table 1). Female participants were generally younger than male and were more likely to have been recruited through a charity. Male respondents had more recently experienced radiotherapy (median 3.5 years post-radiotherapy vs. 5 years for females).

**Table 1:** Sample characteristics

Sample characteristic	No. participants	
	Female (*n* = 14)	Male (*n* = 14)
Recruitment route: *Charity* *NHS site*	11 3	2 12
Age in years, median (range)	59.5 (34–75)	70 (64–82)
Cancer site *Prostate* *Cervix* *Endometrium/uterus* *Anus/rectum*	- 5 5 4	14 - - -
Years since radiotherapy, median (range)	5 (1–17)	3.5 (0.5–7)
Ethnicity *White* *Black*	14 -	13 1
Bowel symptoms self-reported at recruitment *Bowel frequency/urgency* *Bleeding* *Pain* *Diarrhoea* *Excess wind* *Unable to empty bowel* *Tenesmus*	10 2 10 6 8 8 5	9 10 3 4 5 4 4

### Bowel symptoms experienced by participants

Most participants reported multiple symptoms. All bar two reported at least one symptom that featured diminished bowel control, experienced as actual or possible unintended anal discharge or leakage (of faeces, excess wind, mucous, or blood) in the week prior to recruitment (Table 1) and all had experienced diminished bowel control at some point post-treatment. Bowel cramps/pain and constipation were exceptions in that they were not associated with leakage, and in the case of cramps/pain nor were they associated with loss of control. Accounts of symptoms suggested variation in the severity of symptoms as well as type. Bleeding, for example, was experienced by one participant as unproblematic and another as requiring weekly hospital treatment. Cramps for one respondent were ‘mild’ and for another accompanied by sleep disruption and at times, two hours of vomiting.

### Themes

Participants described actions that they took to prevent or reduce their symptoms or to deal with the immediate consequences, and ways in which their daily activities were impacted as a result. We organised these data into four themes: 1) losing control; 2) experiencing embarrassment and fear; 3) managing and reacting; and 4) restriction and withdrawal.

#### Theme 1: losing control

I’ve got no control over it, it just happens and it’s within, literally I get this feeling I need to go to the toilet, and I’ve got to go, I really have to go. And it’s not just minutes, it can be literally seconds… (ID24, male, 74 years, prostate cancer, frequency/urgency, diarrhoea).

I’m incontinent of faeces and that doesn’t necessarily mean that, that’s just diarrhoea so that is probably the biggest issue. (ID09, female, 61 years, anal/rectal cancer, frequency/urgency, excess wind, pain/cramps).

This theme describes experiences of anal leakage or unintentional discharge. These were a common feature of most of the bowel symptoms described by participants. The theme also incorporates experiences of the threat, risk, or possibility of leakage that comes with lost control. Some participants spoke explicitly about ‘control’ or lack of it; several described their bowel or body as an external agent that was exerting control over them.

I’d just like to be a little bit more in control I think of my bowel, rather than my bowel being in control of me. I want to swap it round. I don’t want to be dictated to anymore by my bowel. (ID10, female, 48 years, cervical cancer, frequency/urgency, diarrhoea, excess wind, pain/cramps).

…how the body is now it’s got control of me… (ID01, female, 66 years, endometrial/uterine cancer, excess wind, pain/cramps, feeling unable to completely empty bowels, tenesmus)

The experience of uncontrolled leakage or discharge was also referred to by participants euphemistically as an ‘incident’ or ‘accident’, suggesting that this could be difficult to talk about, or label, and highlighting how these experiences were distinct moments that pulled individuals outside of their everyday/acceptable lived experience and into something untoward or unwelcome.

#### Theme 2: experiencing embarrassment and fear

I’m terrified of going to events with people and ending up with my bowel evacuating. I’m terrified of that, I’m terrified of that, I’m terrified of that (ID06, female 61 years, cervical cancer, frequency/urgency, excess wind, pain/cramps).

…the fear is always there that there will be an embarrassing situation… (ID15, male, 64 years, prostate cancer, frequency/urgency, diarrhoea, excess wind)

This theme describes immediate and short-term psychological consequences of public leakage or the threat of this. The experience of uncontrolled leakage or discharge had negative psychological consequences particularly if it occurred in a public place or there was a possibility of others being aware of it. Participants were embarrassed and/or anxious or fearful at the possibility of being embarrassed. Participants’ accounts of uncontrolled leakage or discharge were often very emotive. Descriptions of the experience included, ‘embarrassing’, ‘terrible’, and ‘awful’, and left participants feeling ‘upset’, ‘desperate’, and ‘distraught’. The experience of actual or potential public soiling left participants with a loss of confidence, anxiety, and fear about the possibility of uncontrolled leakage or discharge occurring in public. Embarrassment was not restricted to leakage but could also be associated with the possibility of other people becoming aware of participants’ need to use a toilet frequently or urgently.

#### Theme 3: managing and reacting

If I’m going to be somewhere…I’ll take an Imodium before I go, even if I haven’t had an upset stomach…it’s just planning ahead, because it’s not nice…having that extra worry… (ID08, female, 34, cervical cancer, frequency/urgency, diarrhoea, bleeding, excess wind, pain/cramps).

…everywhere I go … I think … where’s the toilet, where can I go, what can I do, where have I got to go, how can I get back to the car? And that’s what I do. And that’s really my life now. (ID24, male, 74 years, prostate cancer, frequency/urgency, diarrhoea).

This theme describes actions taken by participants to avoid leakage or discharge and/or to avoid or reduce the extent to which this would become public. Actions could be broadly categorised as those intended to *prevent* leakage/discharge, which included restricting the types or quantities of food consumed, pelvic floor exercises to increase bowel control, and taking medication to slow the passage of food through the bowel, and those intended to prevent leakage or discharge from *causing soiling or becoming public*. The latter included changing the timing of activities to be home at times when loss of bowel control could be expected (often this meant leaving the house later in the day, after bowels had emptied), learning the location of the nearest toilet and ensuring easy access to this, wearing an absorbent pad, and having access to a change of clothes and cleaning wipes.

#### Theme 4: restriction and withdrawal

…on a daily basis it is sometimes up to two hours in the toilet basically just kind of sitting there thinking well you know, the world’s going on around me and I’m stuck in the bathroom. (ID10, female, 48 years, cervical cancer, frequency/urgency, diarrhoea, excess wind, pain/cramps).

… it’s changed my life in respect of what I used to be like. I have no inclination to really go anywhere. That’s the way it’s affected me. (ID26, male, 69 years, prostate cancer, frequency /urgency, bleeding, pain, tenesmus).

This theme describes the largely social consequences of participants’ efforts to avoid or reduce the experience of actual or potential public leakage and discharge. Participants reported imposing restrictions on their eating and on their use of time (for example, not leaving the house until their bowels had emptied) and space (for example, remaining close to known toilets), as described in the ‘Managing and reacting’ section. Participants also reported abstaining from leisure activities that required spending time in public places, reducing sexual contact, and reducing their social circles. These restrictions could also impact on other family members.

[we] cannot go to a restaurant, cannot go out for birthdays … can’t do that, never done that now, for a couple of years now we haven’t done that. And that’s again, that affects my wife rather than me, I miss it, but my wife misses it as well. She doesn’t get to go because what’s the point in celebrating our birthday or our wedding anniversary or something like that when you can’t go out? (ID24, male, 74 years, prostate cancer, frequency/urgency, diarrhoea).

Restriction and withdrawal were not universally reported. A few respondents also described making efforts and/or accepting the risk of public leakage so as not to be restricted.

Am I going to let those sort of incidents actually prevent me from having a life at all? No, I’m not. I’m not going to put my whole life on hold. So, I just get on with things in as normal a way as I can… (ID12, female, 62 years, anal/rectal cancer, frequency/urgency, bleeding, excess wind, pain, tenesmus).

### Working and the workplace

It’s a business isn’t it and nobody seems to care… (ID09, female, 61 years, anal/rectal cancer, frequency/urgency, excess wind, pain/cramps)

The work context presented specific challenges and all four themes were represented. The possibility of losing control and of leakage or discharge occurring was experienced in the workplace (theme 1) and was associated with embarrassment and fear (theme 2). There was also embarrassment associated with discussing symptoms with employers and fear of having to spend excessive time at work in the toilet.

…it was that fear of going into work …will I poop myself today?… will I need to spend hours on the toilet? (ID04, female, 51 years, anal/rectal cancer, frequency/urgency, excess wind, pain/cramps, feeling unable to completely empty bowels, tenesmus).

… I said oh I’ve got some bowel issues and I rolled my eyes, but I mean I didn’t say any more than that. So, it’s quite a hard thing to talk to your boss about. (ID13, female, 51 years, endometrial/uterine cancer, frequency/urgency, pain).

Concerns about being perceived as unprofessional could lead people to focus on managing symptoms themselves (theme 3). Self-management efforts could be helped or hindered by flexibility and support from employers, or lack thereof.

I don’t tell my work about all of these symptoms. I try to manage them myself ‘cos I don’t want them to think any less a person of me because I have all these symptoms and any privileges that I think that I would be entitled to… (ID17, female, 56 years, endometrial/uterine cancer, frequency/urgency, diarrhoea, pain/cramps, feeling unable to completely empty bowels, tenesmus).

Flexibility in timing of work activities was important in facilitating self-management, as was adequate toilet provision. Knowing the location of toilets in the workplace was not sufficient. It was also important to know that these facilities would be vacant when needed, clean, comfortable, and sufficiently private to be used without attracting the attention of colleagues or clients.

I … work from home every now and then if I woke up and things were not great … having that flexibility is so key. (ID08, female, 34, cervical cancer, frequency/urgency, diarrhoea, bleeding, excess wind, pain/cramps).

...if I go to the toilet and somebody else is in it… I have to walk all the way through the office. I’ve called it doing the walk of shame…because if I had a problem everyone would know I’ve got a problem. (ID09, female, 61 years, anal/rectal cancer, female).

Ultimately some participants withdrew from work, with potentially significant psychological and financial consequences (theme 4).

… it had been a job that I loved but I just couldn’t cope anymore…to suddenly not be working and you’re just sat at home thinking what, what do I do? What am I going to do? You’ve got money issues then as well…(ID04, female, 51 years, anal/rectal cancer, frequency/urgency, excess wind, pain/cramps, feeling unable to completely empty bowels, tenesmus).

## Discussion

**Fig. 1:**
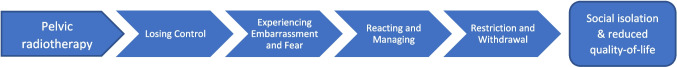
Mapping themes to a pathway from symptoms to quality-of-life impact

This study provides a comprehensive and detailed insight into the bowel problems experienced by survivors post-radiotherapy. We organised our data around four themes: losing control, experiencing embarrassment and fear, managing and reacting, and restriction and withdrawal. These themes suggested a process or pathway by which chronic post-radiotherapy bowel symptoms may have led to social isolation (Fig. 1). Inability to control discharge in public led to experiences of embarrassment and anxiety or fear about potential embarrassment. Participants tried to manage symptoms to reduce or prevent discharge and reacted to conceal this. They also sought to minimise the risk of embarrassment by restricting their social circle, leisure activities, travel outside of the home, and sometimes intimacy with a partner. Lost control led to restrictions on activities and ultimately to increased isolation and reduced ability to fulfil social and work roles.

The findings of this study share common features with those of work on FI. As with FI, the symptoms described by participants in our study feature lost bowel control, experienced either as uncontrolled anal leakage or discharge or the perceived likely possibility of this occurring. A small number of qualitative studies in the USA, not restricted to cancer survivors, have considered the impacts of FI. Chelvanayagam and Norton [14] found that women experienced embarrassment and fear of soiling and made efforts to keep FI hidden. Collings and Norton [15] explored the psychosocial and sexual consequences of FI with women recruited through an FI clinic. These women reported using medication or pads to manage or reduce the possibility of soiling and restricted their activities to reduce the possibility of others becoming aware of their condition. Our participants were cancer survivors, recruited for their experience of chronic bowel symptoms and not specifically FI; however, similar to Ludlow [7], it was the uncontrolled leakage of faeces that elicited the most emotional and negative descriptions from participants.

Following Goffman’s work on stigma [16], Ludlow’s study of affected cancer survivors [7] described the experience of FI as being ‘stigmatising’. Participants in that study believed that they were identified as having a socially unacceptable condition (FI) leading to anxiety about potential embarrassment to which a frequent response was to withdraw from social interactions and to limit activities away from the home. Participants in our study, whose symptoms included lost bowel control, also followed a pattern of withdrawal, avoiding public gatherings, withdrawing from the workplace, and reducing their circles of friends. Some went further, and described avoiding intimacy with partners, highlighting how these symptoms can impact adversely on sexual functioning.

While the concept of ‘stigma’ provides a useful way of understanding experiences with FI, a more fundamental explanation may lie in the way aspects of human behaviour have been shaped through evolution by strategies to avoid disease. Curtis [17, 18], for example, suggested that avoiding faecal contamination provides protection from infection and that social rules develop to protect group members from exposure to this risk. Humans are group living, abiding by social rules is important and embarrassment, or fear of embarrassment can serve to encourage adherence to group norms. Embarrassment has been described as ‘social pain’ [19], encouraging people to avoid potentially damaging social interactions (deviance from socially accepted behaviour) in the way that physical pain encourages avoidance of potentially damaging physical ones.

For the individual experiencing bowel symptoms after pelvic radiotherapy, it probably makes little difference which theoretical basis is employed in their description. The concept of embarrassment as social pain, however, might serve as a potent reminder that the suffering associated with bowel symptoms has a strong social component that needs to be recognised, addressed, and avoided as far as possible. As we show, the bowel symptoms experienced by survivors bring emotional and social consequences which could impact negatively on quality-of-life measures (e.g. [20]), and our findings help explain how and why those experiencing bowel symptoms report reduced quality of life (e.g. [4]).

Participants in our study used various means to manage their symptoms and/or mitigate their consequences. Strategies included dietary changes, exercise, and medication to minimise or prevent leakage, ensuring access to toilets, adjusting activities around bowel patterns, use of pads to prevent leakage into clothing, and use of a change-kit and spare clothing to avoid or conceal public evidence of leakage. Accessing appropriate healthcare support for chronic bowel symptoms post-radiotherapy can be challenging [7]. This makes actions by survivors themselves, with the support of others, including healthcare professionals and family members (i.e. supported self-management), potentially more significant for improving quality of life.

A growing body of literature explores experiences associated with returning to work after cancer. A systematic review [21] listed the experience of anxiety among the psychosocial challenges faced by cancer survivors trying to sustain work roles after return. Taskila and Lindbohm [22] note that when the employer makes efforts to take illness into account in planning and managing work, this improves the ability of returnees to cope. Remnant [23] studied experiences of academics with (non-cancer) gynaecological conditions that resulted in unintended discharge, characterised as ‘leaky bodies’. They observed that managers used a dichotomous definition of the academics’ time as either productive or absent and proposed that this negated the value of time required to manage bodily discharge to comply with institutional norms and within the constraints of institutional provision of washroom facilities. While these studies did not specifically refer to bowel symptoms, our study outlined how the experience of bowel symptoms can lead to anxiety and ultimately to withdrawal from the workplace, and the consequences this can have for individuals in terms of social isolation and lost income. Our participants reported that having flexibility in the timing of work activities can be key in reducing anxiety and facilitating a sustained return to work. Lack of flexibility was a cause for anxiety and a contributing factor in decisions to withdraw from work.

In their study of women with FI, Peden-McAlpine [9] reported that fear of humiliation that would result from soiling at work restricted women’s engagement in productive work outside of the home. Our study found that anxiety and embarrassment went beyond possible soiling and included being seen to use toilets more frequently, urgently, or for longer than others. The work context was therefore particularly challenging in relation to the strategies used by participants to avoid embarrassment. Availability of adequate toileting and washing facilities could not always be guaranteed, and their location may not have been sufficiently private, or sufficiently close for urgent access. Despite many of the experiences described occurring subsequent to the UK’s 2010 Equality Act, and the obligations it placed on employers to accommodate the needs of employees returning to work after cancer, there were substantial differences in the responsiveness of employers to their employees’ needs.

Comparing the workplace and home contexts and guided by a social relational model of disability, Remnant [23] argues that the additional challenges presented by the workplace do not arise inevitably from the experience of symptoms but are barriers, actively created by policies and practices that fail to acknowledge diversity in body functioning. Seen from this perspective, theme 4 of our model might better be labelled ‘restriction and *exclusion’*. Casting the experience as ‘withdrawal’ perhaps reflects the extent to which the dominant, medical model of disability, framed both participant accounts and subsequent analysis.

### Implications

Our findings underline the importance of identifying affected individuals and carrying out appropriate ‘screening’ so that treatments can be offered to mitigate symptoms [24]. Mitigation efforts need to be cognisant of, and address, the effects of embarrassment in addition to the practicalities of managing and living with symptoms. Despite some of the very powerful quotes from participants, negative impacts were not uniform and there may be learnings from individuals with less negative experiences that could be applied to help mitigate symptoms and impacts for others. These might include practical strategies such as titration of medication dose as well as psychological strategies for managing anxiety and embarrassment associated with potential public leakage or discharge. Future work might explore this.

In the workplace, support needs to not only ensure toilets are readily accessible but also recognise the importance to survivors of avoiding embarrassment or the risk of embarrassment. This requires the security of always being able to access a toilet discretely and without delay as well as flexibility with work arrangements if symptoms temporarily worsen.

The impacts of chronic bowel symptoms on the daily lives of participants in this study point to a need for development of effective self-management interventions for this survivor population. Self-management is known to face challenges for sustained uptake [11] and the design of interventions would benefit from the inputs of survivors and an understanding of their experiences. Future work might explore existing self-management strategies in more depth, seeking ways to build on these based on the experiences of survivors and health professionals.

### Limitations

While this is (as far as we are aware) the largest qualitative study of cancer survivors experiencing bowel symptoms following pelvic radiotherapy, and one of the few qualitative studies of FI to include both men and women, it is important to recognise the possible effects of our sampling and recruitment strategy on the data collected. Recruitment of volunteers through cancer charities may have identified individuals who were more actively engaged with issues around cancer survivorship and/or who had faced significant challenges and felt able to articulate these. Those recruited through charities tended to be younger women (most likely a function of the fact a cervical cancer charity very actively supported recruitment). Our subsequent recruitment through UK NHS hospitals specifically targeted men as well as those with experience of rectal bleeding. These respondents were often more reticent and sometimes less troubled by their symptoms. While this may reflect gender differences in comfort in talking frankly about such personal issues and/or participants’ life stages, it may also be due to the larger irradiated volume in the treatment of gynaecological cancers and the greater attendant risk of unwanted consequences of radiotherapy. Our sample was sufficient to reflect a range of experience with chronic bowel symptoms but was not intended to allow in-depth comparisons of sub-groups, based for example on gender or symptom-type. Similarly, although we identified experiences at work as an important element of our data, exploring this issue in depth was not a principal aim of our study and some of our respondents (particularly the men with prostate cancer) were already retired at the time of treatment. Our sample also lacked ethnic diversity, being restricted to English language speakers, all but one of whom self-identified as White.

## Conclusions

Our findings add to a growing body of evidence that the consequences of radiotherapy can have lasting and negative impacts on survivors’ quality of life. These impacts can be severe, leading to survivors’ withdrawal, to differing extents, from society, leaving them less able to fulfil previous roles, and bringing emotional and, in some instances, financial consequences. Survivors employ a variety of methods and strategies in living with their symptoms. Some of these facilitate continued role fulfilment but some constitute withdrawal from pre-treatment roles. Current healthcare provision and statutory protections fail to fully meet the needs of people following pelvic radiotherapy, suggesting a need to develop and implement more widespread, evidenced, and supported self-management, designed in collaboration with those affected.

## Supplementary Information

Below is the link to the electronic supplementary material.

Supplementary file1 (DOCX 43 KB)

## Data Availability

The anonymised transcripts analysed during the current study may be available from the corresponding author upon reasonable request for a period of 5 years following the end of the study.
