# Supplementary material for: From pelvic radiation to social isolation: a qualitative study of survivors’ experiences of chronic bowel symptoms after pelvic radiotherapy
Source: J Cancer Surviv. 2024 Jan 6;19(3):1019–27. doi: 10.1007/s11764-023-01527-6 (PMC12081541; doi:10.1007/s11764-023-01527-6)
Supplement: Supplementary file 1 — Supplementary file1 (DOCX 43 KB) [file 11764_2023_1527_MOESM1_ESM.docx]

**Online Resource 1: Additional illustrative quotes**

**From Pelvic Radiation to Social Isolation: A Qualitative Study of Survivors’ Experiences of Chronic Bowel Symptoms after Pelvic Radiotherapy.**

Journal of Cancer Survivorship

**Authors**

Biran^1^, Dobson^1^, Rees^1^, Brooks-Pearson^2^, Cunliffe^3^, Durrant^4^, Hancock^5^, Ludlow^6^, Neilson^7^, Wilson^8^, Sharp^1^

**Affiliations**

^1^Population Health Sciences Institute, Newcastle University, Newcastle upon Tyne

^2^Northern Centre for Cancer Care, The Newcastle upon Tyne Hospitals NHS Foundation Trust

^3^South East London Cancer alliance, London

^4^Somerset NHS Foundation Trust, Taunton

^5^North Tees and Hartlepool NHS Foundation Trust

^6^Cardiff & Vale University Health Board

^7^Department of Gastroenterology, South Tyneside and Sunderland NHS Foundation Trust, South Shields

^8^The Royal Marsden NHS Foundation Trust, London

**Corresponding author**

Linda Sharp, Linda.Sharp@newcastle.ac.uk

**Additional illustrative quotes organized by theme**

| **Quote** |  | **Participant Characteristics** | |
| --- | --- | --- | --- |
| **Theme 1: Loss of Control** | | | |
| …how the body is now…it’s got control of me. | | | ID01, female, 66 years, endometrial/uterine cancer, excess wind, pain/cramps, feeling unable to completely empty bowels, tenesmus |
| It’s like when you’re a kid and your parents get really annoyed with you when you’re going on a car journey ‘cos you suddenly need the toilet. It’s like that. I don’t have much ability to control it. | | | ID05, female, 40 years, cervical cancer, frequency/urgency, pain/cramps, diarrhoea, feeling unable to completely empty bowels |
| …you know, are you going to poo yourself…? | | | ID06, female 61 years, cervical cancer, frequency/urgency, excess wind, pain/cramps |
| I was about a mile from the house…and my bowels just evacuated themselves. Luckily I had a waterproof jacket on and dark pants...I had to go back home, completely evacuated. Luckily didn’t meet anybody and that happened numerous times. | | |  |
| I’m incontinent of faeces and that doesn’t necessarily mean that, that’s just diarrhoea so that is probably the biggest issue. | | | ID09, female, 61 years, anal/rectal cancer, frequency/urgency, excess wind, pain/cramps |
| if I need to go to the toilet, I need to go… | | |  |
| … last week me and my husband, and we were talking to our son on the phone and I knew I needed to go to the loo and stupidly didn’t go right away and had an accident in the hotel room. Luckily I was wearing my big knickers and it was fine you know took them off, cleaned myself up, got sorted but I end up then crying. | | | ID10, female, 48 years, cervical cancer, frequency/urgency, diarrhoea, excess wind, pain/cramps |
| I’d just like to be a little bit more in control I think of my bowel, rather than my bowel being in control of me. I want to swap it round I don’t want to be dictated to anymore by my bowel. | | |  |
| I just want to get to the stage where I’ve got more control, whether it’s psychological or physical I think probably a bit of both. | | | ID11 female, 65 years, anal/rectal cancer, frequency/urgency, diarrhoea, bleeding, pain, excess wind, feeling unable to completely empty bowels |
| I have to go to the toilet… that’s like, I have to go, there would be an urgency and I would have to get there… you know, there and then, so there’s no wait time. | | | ID13, female, 51 years, endometrial/uterine cancer, frequency/urgency, pain |
| bowel movements are the real problem … if I have to go, I really do have to go. | | | ID20, male, 64 years, prostate cancer, bleeding |
| wind, which I have no control over…it just pops out. I can’t stop it. … | | | ID22, male, 66 years, prostate cancer, frequency/urgency, bleeding, excess wind |
| I’ve got no control over it, it just happens and it’s within, literally I get this feeling I need to go to the toilet and I’ve got to go, I really have to go. And it’s not just minutes, it can be literally seconds, | | | ID24, male, 74 years, prostate cancer, frequency/urgency, diarrhoea |
| …one Sunday morning I was stuck for something like 30 minutes standing still, unable to move literally, ... I was pretending to use the phone because I dare not move, I dare not move. And then when I did, I didn’t make it, I soiled myself, I couldn’t do anything but. And it’s awful, it is diarrhoea. It’s virtually liquid and it’s terrible, terrible thing to happen. | | |  |
| I was sitting in the car… having a coffee and reading the paper. All of a sudden I just felt this overriding urgency. I actually got out of the car to try and alleviate it, but it didn’t, and I just could feel my bowels opening into my underwear. That was the first time, and I had to get home and get changed, because I had to change everything. Trousers, underwear, and I noticed in the faeces was quite a lot of mucus and some blood, clotted blood…I felt quite distraught. I thought, what’s happening here?... I remember …phoning the cancer care coordinator. I was sitting in the car, and it was happening again, and what I said to her was I’m desperate here. I’m desperate for some help, because it’s just happening all the time. … that was my very words … I’m desperate here, I just can’t go on like this. … it was a very emotional call … | | | ID26, male, 69 years, prostate cancer, frequency /urgency, bleeding, pain, tenesmus |
| I have had one accident. I was going up to Northumberland, and it started and I just couldn’t stop it. It was terrible, you can imagine… I was in the car going to Northumberland, and I got the urge, I got the feeling and it just started, and I just couldn’t do anything about it. | | | ID29, male, bleeding – controlled PuraStat, urgency |
| I have had one accident. I was going up to Northumberland, and it started and I just couldn’t stop it. It was terrible, you can imagine… I was in the car going to Northumberland, and I got the urge, I got the feeling and it just started, and I just couldn’t do anything about it. | | |  |
| **Theme 2: Experiencing Embarrassment and Fear** | | | |
| …I’ve got to go...I’ve just got to go, so I am worried about it in social situations. | | | ID05, female, 40 years, cervical cancer, frequency/urgency, pain/cramps, diarrhoea, feeling unable to completely empty bowels |
| …people don’t really understand when your bowels want to move and they just go woosh out of you. Its sort of erm you know you’re sort of in shock for a couple of days you know it was hard to describe it. You don’t ever think that’s going to happen to you, and its, it’s a really, it’s a discomfabulating experience…you go into sort of [another] world, is this really happening to me. …you don’t know what real and what’s not real… | | | ID06, female 61 years, cervical cancer, frequency/urgency, excess wind, pain/cramps |
| If I start to soil myself I’d be just devastated and that’s what would happen to me if I’m not besides a toilet | | |  |
| If I go out I’m always worried about can I go to the loo | | | ID10, female, 48 years, cervical cancer, frequency/urgency, diarrhoea, excess wind, pain/cramps |
| if I need to sit on the loo, it’s not just a case of just going to the loo. I then have to make sure that I’m clean … I clean myself and use wipes or get in a bath or a shower, whatever to sort myself out so you know I could be gone for twenty minutes just going to the loo whereas anyone else can just nip to the loo and be back. So that affects my work life. | | |  |
| … it’s a confidence thing, you know, are you going to poo yourself, are you going to wet yourself? | | | ID11 female, 65 years, anal/rectal cancer, frequency/urgency, diarrhoea, bleeding, pain, excess wind, feeling unable to completely empty bowels |
| it’s alright your colorectal nurse saying ‘have a jungle poo’, I’d be absolutely mortified… | | |  |
| …it’s embarrassing, it’s horrible, it’s humiliating… | | | ID12, female, 62 years, anal/rectal cancer, frequency/urgency, bleeding, excess wind, pain, tenesmus |
| sometimes that’s like I have to go, there would be an urgency and I would have to get there…there’s no wait time…I haven’t had any accidents…that would be horrific. | | | ID13, female, 51 years, endometrial/uterine cancer, frequency/urgency, pain |
| …the fear is always there that there will be an embarrassing situation. | | | ID15, male, 64 years, prostate cancer, frequency / urgency, diarrhoea, excess wind |
| I had an incident when I was away on holiday. I was incontinent and I saturated the whole bed, and it was really embarrassing… It was really very embarrassing. | | | ID17, female, 56 years, endometrial/uterine cancer, frequency/urgency, diarrhoea, pain/cramps, feeling unable to completely empty bowels, tenesmus |
| I got points on my driving license because I was speeding to get to a toilet and I was stopped and I was so embarrassed I didn’t want to say… I’m going to be incontinent. I ended up being incontinent and getting upset. | | |  |
| you can take medication and you can amend your diet but you’re still going to live with this fear that, like when I went away on holiday and I was incontinent in the middle of the night, well that could happen to me again. | | |  |
| I was tending to have it in me mind oh if I go out this [uncontrolled bleeding] is going to happen, that’s going to happen. I never think of it now. | | | ID19 male, 79 years, prostate cancer frequency/urgency, bleeding, pain/cramps, tenesmus |
| it was rather embarrassing when you’re walking round the town or getting out the car and everything’s stained up. | | | ID22, male, 66 years, prostate cancer, frequency/urgency, bleeding, excess wind |
| very nervous to go anywhere. So that’s really what I would class as the main symptoms for it that you just didn’t feel you could let’s say go to the pictures or go to the pub and if you weren’t dressed accordingly to try and contain a certain amount of it, it was embarrassing. | | |  |
| it’s very embarrassing, you know, when you’re next to people and it just comes out | | | ID23 male |
| one occasion which was really, really embarrassing, I had someone here mending something, … I ended up in the toilet and I was shouting down to the guy, OK just leave it, I’ll sort the money out with your boss when I get round there, he went out and closed the door and he must have thought I was a blinking idiot and I was in the toilet and really I just couldn’t get out the toilet. And the guy was shouting, are you all right Mr-? I’m fine, yes, OK, yes just leave me with it, just leave me alone please and just close the door behind you. | | | ID24, male, 74 years, prostate cancer, frequency/urgency, diarrhoea |
| I may soil my trousers or something like that…. It really is an awful feeling... the thought of that happening when I’m out somewhere … you know, it’s frightening really, it is it’s terrifying. | | |  |
| **Theme 3: Managing and Reacting** | | | |
| I don’t eat oranges or acidy stuff…I don’t eat oranges cos you’ve got pips that doesn’t break up, and not eating nuts, I like nuts, you can’t eat nuts because that’s fibre to digest, …I have porridge, I have shredded wheat, I have a mixture of cornflakes and shreddies and that’s just having a little bowl and not having a lot. | | | ID01, female, 66 years, endometrial/uterine cancer, excess wind, pain/cramps, feeling unable to completely empty bowels, tenesmus |
| [I drink]…lactose-free milk…decaf coffee, I have to be very careful when I’m eating salads…pastry is another one…if now and again I really fancy it, I definitely know I’m not going anywhere…… | | | ID04, female, 51 years, anal/rectal cancer, frequency/urgency, excess wind, pain/cramps, feeling unable to completely empty bowels, tenesmus |
| I have to carry a bag around with me…like a little change bag but erm but that’s just life now erm to be honest…always have a spare change of clothes and wipes and everything else. | | |  |
| I need to take Imodium if I go anywhere… | | | ID05, female, 40 years, cervical cancer, frequency/urgency, pain/cramps, diarrhoea, feeling unable to completely empty bowels |
| I know not to plan to be going anywhere within the first hour or two of getting up in the morning. | | |  |
| … very acidic food, orange juice and stuff like that, no way, grapefruit no, or lots of fatty things… spicy things…[ these are] strategies, it’s liveable. | | |  |
| I have to be careful now and not eat too much so I’ve tried to push my plate away because I love me dinner, I love my proper food and I have to try…as soon as I be a little bit full, push it away and not to nibble. | | | ID06, female 61 years, cervical cancer, frequency/urgency, excess wind, pain/cramps |
| If I’m going to be somewhere…I’ll take an Imodium before I go, even if I haven’t had an upset stomach…it’s just planning ahead because it’s not nice…having that extra worry… | | | ID08, female, 34, cervical cancer, frequency/urgency, diarrhoea, bleeding, excess wind, pain/cramps |
| I did have a conversation with the bosses and work from home every now and then if I woke up and things were not great | | |  |
| I would probably take some medication…if I’d got diarrhoea or if I’d gone to the toilet sort of maybe four times I’d be starting to think I need to be calming this down… and that would sort of allow me to be able to go out… | | | ID09, female, 61 years, anal/rectal cancer, frequency/urgency, excess wind, pain/cramps |
| we have in our office, one disabled toilet that is actually in that office but in the building, there are other toilets but they’re further away and there is another disabled toilet as well right down the other end of the building so for me if I need to go to the toilet, I need to go. Erm, so if I go to the toilet and somebody else is in it, | | |  |
| I really need a disabled toilet because they have wash facilities … people don’t understand or they’re not willing to try and understand….a lot of people think that if you’re disabled you’re in a wheelchair...I just don’t think work have been that helpful in respect of the toilet at all. | | |  |
| I can’t eat pork for example, and I love pork. So if I eat pork I have to make sure that I’m not going anywhere afterwards, that I’m indoors. So yeah, certain foods erm I can’t eat a lot of vegetables, a lot of, if I have brown bread I can’t have it sort of every day, it has to be sort of every other day. So yeah dietary really and just the loperamide is really how I’ve had to manage it, because there’s not a lot else really I can do. | | | ID10, female, 48 years, cervical cancer, frequency/urgency, diarrhoea, excess wind, pain/cramps |
| If I go out … I’ve always got kit with me. | | |  |
| dietary really and just the loperamide is really how I’ve had to manage it, because there’s not a lot else really I can do. | | |  |
| I fought tooth and nail just to get them to put a shelf in the bloomin’ disabled toilet for me so I could change my bag while I was at work, cos I had nowhere to put anything, I was having to put stuff on the floor, which obviously you don’t want to be doing. | | |  |
| … if I need to sit on the loo, it’s not just a case of just going to the loo. I then have to make sure that I’m clean … I clean myself and use wipes or get in a bath or a shower, whatever to sort myself out so you know I could be gone for twenty minutes just going to the loo whereas anyone else can just nip to the loo and be back. So that affects my work life. | | |  |
| …the phone’s constantly, constantly going, you end one call you start another. So to go to the loo you have to kind of log out, so I feel I always have to explain I may be longer, I may need an 8-10 minute comfort break… | | |  |
| I’m trying not to control it by loperamide because when you read the causes of bowel cancer… I’m trying not to take any medication that I don’t have to, and I really don’t want to mess around with my bowels by controlling it with loperamide, or if it goes the other way which it can occasionally I don’t want to take laxative unless I really have to. | | | ID11 female, 65 years, anal/rectal cancer, frequency/urgency, diarrhoea, bleeding, pain, excess wind, feeling unable to completely empty bowels |
| I’m trying to control it by diet, I’m doing a food diary to see what affects it and what doesn’t. | | |  |
| if I go out for a meal…I have to know where the toilets are, I have to know my escape route… whenever I go anywhere…I know where every toilet is in every supermarket…and all routes in between…if I got on a train I’d have to know as soon as I got on where the toilets were and I’d be in a carriage near the toilets…if I go to the theatre I’d rather have an aisle seat so I can get out quick…it does affect most things… We might do a seven mile walk round [name of town] but I know where every single toilet is where I go. | | |  |
| I’ve always known about the pelvic floor exercises and I’ve always generally done them intermittently, … I’m not sure whether it was in this MacMillan book or whether it was in the information that PRDA sent me about … try and train your bowels. … so one of the exercises is to just sometimes try not to go immediately, to try and train your bowels… So It works sometimes but not all the time, so I am doing that type of exercise, and I had add pelvic floor exercises more regularly. | | |  |
| I find still that if I get a bad patch, … where I’m getting a lot of diarrhoea … and total lack of control, … if I purposely try and eliminate [fat] for two or three days, then usually things get a bit more stable. | | | ID12, female, 62 years, anal/rectal cancer, frequency/urgency, bleeding, excess wind, pain, tenesmus |
| a lot of liquid going into the body, and I know that that is going to stimulate the bowel. So one thing that I do do is not drink when I’m eating. It doesn’t always stop me having a problem after lunch, but it sometimes does. | | |  |
| often wear a pad, so long as it’s not dramatic I can control it. Well, I wouldn’t say I control it….I mean sometimes it’s just a bit of soiling on the pad…when I’ve got watery diarrhoea… I’ve got no control over it then…and there’s nothing I can do about that…it’s just really difficult. | | |  |
| most of my contact [at work] was for one person and then another person and another person. So in between each person I was seeing there was an opportunity to rush to the loo if I needed to. So, in that way I was able to manage it. | | |  |
| while I’m at home and pottering about under my own timetable I can manage the symptoms myself…But I’ve got to go back to work… I’ve got half an hour drive to get to work, I’m a teacher, you can’t run out of classroom at the drop of a hat really. | | | ID13, female, 51 years, endometrial/uterine cancer, frequency/urgency, pain |
| I’m going to see the dentist next week, I’ve got a 9.40 appointment and I’ll probably get up very early that morning to kind of make sure I’m ok before I set out. | | | ID14, female,63 years, endometrial/uterine cancer, tenesmus |
| it makes travel difficult so I would deal with that by taking Imodium and taking it prophylactically to avoid the risk of it happening. | | | ID15, male, 64 years, prostate cancer, frequency / urgency, diarrhoea, excess wind |
| last night I’d had wet wind and I’d taken Imodium so I know I’m not going to have a bowel motion today, so that gives me a little bit more freedom. But then tomorrow I have to think well, my bowel’s going to move and am I going to have diarrhoea. | | | ID17, female, 56 years, endometrial/uterine cancer, frequency/urgency, diarrhoea, pain/cramps, feeling unable to completely empty bowels, tenesmus |
| I can’t go out without taking a change of clothes with me…a simple backpack with a change of clothes and wipes and underwear. And that’s what I have to carry with me. | | |  |
| you figure out where you can go to the toilets it sort of gives you a little bit of security. | | |  |
| I’m very lucky that where my office is and where I work the toilet is very close. Now they were getting some work done and the toilet wasn’t, you know they were doing, the toilet was being reconfigured and they were making a bigger disabled toilet, and it was all due to a bigger reconfiguration. So during that period when I knew I didn’t have access to it I just worked from home. | | |  |
| There’s one toilet in the school, it’s off the main school hall so if anyone’s in the school hall, or if anyone’s sitting down in assembly or something, you’ve got a toilet attached to the hall, it’s not very private, and the back wall of the toilet is the side wall of the head’s office. So those things are causing me some anxiety … | | |  |
| … I don’t tell my work about all of these symptoms, I try to manage them myself cos I don’t want them to think any less a person of me because I have all these symptoms and any privileges that I think that I would be entitled to… | | |  |
| I would put some incontinence pads on just in case I had an incident. | | | ID19 male, 79 years, prostate cancer frequency/urgency, bleeding, pain/cramps, tenesmus |
| I carry pads and pants with me. | | | ID22, male, 66 years, prostate cancer, frequency/urgency, bleeding, excess wind |
| I tried, well I’ll stop eating tomatoes and I’ll try this and I was reading about it and I stopped eating chocolate | | | ID24, male, 74 years, prostate cancer, frequency/urgency, diarrhoea |
| I’m taking the diarrhoea tablets in one form or another. | | |  |
| [The GP] …prescribed me loperamide and now I take those. But I don’t take them every day right, my experience with them is the same as Imodium, they make me constipated and sometimes that’s worse when you do want to go to the toilet, you really need to go, you know what I mean? So I’m caught sometimes between [unclear 0:29:12] loperamide and then I’m constipated and then when I do need to go I cannot go today because I’m constipated and I don’t know if I’m going to be loose in half an hour or something and that’s the way I am. So I haven’t yet worked out from a personal point of view what I do with loperamide, do I take them every day, do I take two, do I take one? I’m trying to find out. | | |  |
| …everywhere I go … I think … where’s the toilet, where can I go, what can I do, where have I got to go, how can I get back to the car? And that’s what I do. And that’s really my life now. | | |  |
| I don’t eat as much as I probably should. | | | ID26, male, 69 years, prostate cancer, frequency /urgency, bleeding, pain, tenesmus |
| …. I also carry a bag with me, … which has clean underwear in … wherever I go I always have a bag with a change of underwear. | | |  |
| I make sure I’ve got clean underwear, and I make sure … that I put a pad on. It’s part of normal life now… where up until January I would never have dreamed of wearing a pad. It’s a safety net for me to wear the pad, but I’m still reluctant to do much, to go far afield, because of what could happen with this bowel issue. | | |  |
| I wear them (nappies) throughout the day, because I don’t want to take any chances on leakage either soiling my clothes, or even spoiling what I might be sitting down on | | |  |
| I still wore the nappies just as a safety measure, but I needn’t have bothered. | | | ID27, male, 78 years, prostate cancer, bleeding |
| I had to be aware of where the nearest disabled toilet was. Because it was a matter of getting undressed to clean myself up sometimes, because there was so much blood being lost. | | |  |
| African food tends to be spicy, so I’ve cut down on that. Not 100%, because then I would be craving it. … I’ve cut down on that, not because the doctors told me to, but because I feel that it may be a factor. | | | ID28, male, frequency/urgency, bleeding, diarrhoea |
| before I go out, I make sure that I’ve already had my bowel movement, so I don’t run into any problems outside. | | |  |
| **Theme 4: Restriction and Withdrawal** | | | |
| I gave up work … because I just couldn’t do it anymore. Because if you realise that, if you’ve got issues where you have to keep going to the toilet… [you] need a nice clean toilet that’s not out in the cold…if somebody’s in my loo where do I go? | | | ID01, female, 66 years, endometrial/uterine cancer, excess wind, pain/cramps, feeling unable to completely empty bowels, tenesmus |
| I could be on the toilet for hours and then sheer thought just going in and not knowing what was going to happen… we had one toilet we shared men and ladies …and it was just too much, too much worry, too much stress, even getting up in the morning, you know it was just that fear of going into work…and it had been a job that I loved but I just couldn’t cope with it anymore. | | | ID04, female, 51 years, anal/rectal cancer, frequency/urgency, excess wind, pain/cramps, feeling unable to completely empty bowels, tenesmus |
| It's very, very, hard. My daughter said to me Sunday…oh can we do something today and I said I’m really sorry, I can’t…oh you never can…you’re this you’re that…I said I’m really sorry it’s not my fault you know, I hadn’t slept for 3 nights, I wasn’t going to drive anywhere. I was not in a fit stated to drive so you know it impacts the family too. 99 percent of the time my girls are really, really, understanding but every once and a while it’s you know they get frustrated you know, as I do. Erm and it is totally understanding but it’s impacted their lives as well. | | |  |
| It affects things like sex, because I don’t know…it sounds disgusting…I don’t know if I’m going to mess myself you know if we’re…if we’re having sex so you tend not to | | |  |
| I’m not going to be housebound like a lot of people allow themselves to become…I don’t allow myself to be prevented, I think some people do allow themselves to be …at the end of the day am I going to let those sort of incidents actually prevent me from having a life at all? No I’m not. I’m not going to put my whole life on hold. So I just get on with things in as normal a way as I can,…if it does mean I find myself having to dive behind the bushes or wall when I’m out walking, well that’s just too bad isn’t it, I just get on with things. Yes these things are very debilitating but they don’t stop me, just because I won’t let them. | | | ID12, female, 62 years, anal/rectal cancer, frequency/urgency, bleeding, excess wind, pain, tenesmus |
| I’m virtually a recluse if you like, and I don’t want to be like that. ….I tend not to go out now… | | | ID24, male, 74 years, prostate cancer, frequency/urgency, diarrhoea |
| I used to go to the cinema regularly, but I just stopped doing it because it wasn’t enjoyable. Because I could be sitting there and it would happen, and then I would have to go into the toilets to clean up… we used to go out most days, but that’s something I’m reluctant to do now …I’m a season ticket holder at [name of town] Football Club, and I’ve missed some games because when I’ve gone to the match this has happened, and then I’ve got to leave and go downstairs, and then come back up. The facilities there in football grounds are not great to clean yourself up… and there’s no washing … And you’ve got crowds of people there, and it’s … embarrassing that you’ve got to try and do this. To be quite honest, it’s restricted my ability to do a lot of things… since this has happened, I haven’t been going out as much. It … has affected my life in respect of going out. … I feel as if I’m not going out as much as I used to, or enjoying life as much as I used to... I think it’s to do with the way I feel about things. I feel sort of restricted in what I can do. … it’s changed my life in respect of what I used to be like. I have no inclination to really go anywhere. That’s the way it’s affected me. | | | ID26, male, 69 years, prostate cancer, frequency /urgency, bleeding, pain, tenesmus |
